# Fungal secondary metabolite dynamics in fungus–grazer interactions: novel insights and unanswered questions

**DOI:** 10.3389/fmicb.2014.00788

**Published:** 2015-01-13

**Authors:** Marko Rohlfs

**Affiliations:** Johann-Friedrich-Blumenbach Institute of Zoology and Anthropology, Georg-August-University GöttingenGöttingen, Germany

**Keywords:** *Aspergillus*, chemical defense regulation, fungivory, inducible resistance, secondary metabolites

## Abstract

In response to fungivore grazing fungi are assumed to have evolved secondary metabolite-based defense mechanisms that harm and repel grazers, and hence provide a benefit to the metabolite producer. However, since research into the ecological meaning of highly diverse fungal secondary metabolites is still in its infancy, many central questions still remain. Which components of the enormous metabolite diversity of fungi act as direct chemical defense mechanisms against grazers? Is the proposed chemical defense of fungi induced by grazer attack? Which role do volatile compounds play in communicating noxiousness to grazers? What is the relative impact of grazers and that of interactions with competing microbes on the evolution of fungal secondary metabolism? Here, I briefly summarize and discuss the results of the very few studies that have tried to tackle some of these questions by (i) using secondary metabolite mutant fungi in controlled experiments with grazers, and by (ii) investigating fungal secondary metabolism as a flexible means to adapt to grazer-rich niches.

## INTRODUCTION

Fungal metabolism is an important source of an apparently endless diversity of organic compounds which are not obviously required for the producer’s normal growth and metabolism – one reason why they are called secondary metabolites (SMs). This chemical diversity raises a pertinent yet hardly addressed basic research question in fungal biology: why are SMs produced in such variety, especially since the SM biosynthesis is encoded in clusters of genes whose transcriptional activation is embedded in a tightly regulated network comprising the activity of signaling pathways, epigenetic regulators, fungal hormones, and cellular secretion processes (Tsitsigiannis and Keller, 2007; Roze et al., 2011; Brakhage, 2013)?

Because many fungal SMs are toxic to other organisms, in particular animals, one line of arguments follows the idea that fungi produce specific SMs as direct defense compounds to achieve protection from grazers foraging for nutrient-rich food sources (e.g., Janzen, 1977; Martin, 1979; Camazine et al., 1983; Vining, 1990; Gloer, 1995; Spiteller, 2008). Putative direct defense compounds have emerged primarily due to pharmacological evidence for their deleterious effects on grazers. This evidence stems from studies that report relationships between fungivore mortality, decreased fecundity, and feeding on purified compounds mixed into artificial diets or on fungi expressing the proposed chemical defense trait constitutively (e.g., Wright et al., 1980; Paterson et al., 1987; Panigrahi, 1993; Gloer, 1995; Rohlfs and Obmann, 2009). Such studies provide at best weak correlative evidence as they do not evaluate whether these compounds increase fungal fitness under grazing pressure, which is the defining criterion of a chemical compounds-based defense.

In a series of recent studies (see below), the use of genetically modified fungi manipulated in the expression of candidate defense gene products has corroborated the relationship between SM regulatory mechanisms and resistance against grazers, yet manipulation of pathway-specific defense products have revealed conflicting results. Moreover, first studies have demonstrated complex chemical changes in fungi after fungivore attack, which could in part be related to changes in the capacity to resist grazing. The present review intends to provide a critical discussion of these recent findings and stimulate more research into the ecological causes and consequences of fungal chemical diversity.

## INVESTIGATING FUNGAL RESISTANCE WITH GENETICALLY MANIPULATED FUNGI

Since more than a decade, functional genetic approaches have provided unparalleled insights into how fungi regulate their chemical diversity. Despite the availability of an increasing number of well–controlled mutant fungi, functional evaluations have largely been restricted to biochemical and medical aspects of fungal biology. Very few studies have used genetically modified fungi to conduct functional analyses of SM genes in the context of interactions with grazers.

### GLOBAL SM REGULATION

Six of these studies focused on *Aspergillus* spp. *laeA* loss-of-function mutants in interactions with different soil arthropods, Collembola, (Rohlfs et al., 2007; Janssens et al., 2010; Staaden et al., 2011; Stötefeld et al., 2012) or facultative fungivorous *Drosophila melanogaster* larvae (Trienens et al., 2010; Caballero Ortiz et al., 2013). The methyltransferase-domain protein LaeA is essential for both appropriate developmental processes coupled with the biosynthesis of various SMs, including mycotoxins, such as sterigmatocystin (Bok and Keller, 2004; Sarikaya Bayram et al., 2010). Chemical deficient Δ*laeA Aspergillus* sp. mutants generally are less detrimental to grazer fitness than wild type fungi (reduced mortality, enhanced reproductive output) and, when given the choice, fungivores prefer grazing on the Δ*laeA* mutants. The latter appears to be mediated by a combined effect of differences in both the non-volatile SM and the volatile chemical profile (Staaden et al., 2011; Stötefeld et al., 2012). The LaeA-dependent capacity to harm insect grazers seems to be fungal species-specific though: while Δ*laeA Aspergillus nidulans* almost entirely lost the potential to kill fruit fly *D. melanogaster* larvae, Δ*laeA A. flavus* and Δ*laeA A. fumigatus* were still fatal to the insects, yet the onset of larval mortality was significantly delayed (Trienens et al., 2010). Importantly, compared to wild type, the lack of expression of *laeA* in all fungi tested increased their susceptibility to grazer damage (Rohlfs et al., 2007; Trienens et al., 2010; Stötefeld et al., 2012).

Loss of the capacity to kill fruit fly larvae was also evident for *A. nidulans* impaired in the expression of VeA (Trienens and Rohlfs, 2012), which indicates that an intact velvet (VelB-VeA-LaeA) complex (Bayram et al., 2008) is required for building resistance against grazers. Therefore, global regulatory mechanisms controlling both SM diversity and development have a distinct function in mediating protection from fungivore attack, and I would predict a similar role of these mechanisms in fungi beyond *Aspergillus*.

### PATHWAY-SPECIFIC SM REGULATION

A review of the literature indicates that there are only five published studies that have used pathway-specific mutant strains to test for the influence of single SMs on the outcome of interactions with fungivores (Scheu and Simmerling, 2004; Staaden et al., 2010; Trienens and Rohlfs, 2012; Yin et al., 2012; Cary et al., 2014). As demonstrated by the use of knock-out mutants in feeding assays with Collembola, *Folsomia candida* and *Protaphorura armata*, and the nitidulid beetle *Carpophilus freemani*, polyketide synthase-driven formation of pigments dihydroxynaphthalene melanin in *A. fumigatus* conidia and asparasone A in sclerotia of *A. flavus* proved to impair or deter grazers, respectively, (Scheu and Simmerling, 2004; Cary et al., 2014). The latter is of particular interest because it is the first demonstration of a grazer-deterring compound whose biosynthesis is confined to fungal tissue that has a special function in ensuring survival under unfavorable conditions. However, how such pigments contribute to directly repelling grazers is unknown.

The polyketide sterigmatocystin is a characteristic mycotoxin of *A. nidulans*, which has insecticidal properties (Chinnici et al., 1983; Rohlfs and Obmann, 2009). In accordance with the proposed role of sterigmatocystin in mediating resistance against grazers, Collembola avoided feeding on a mutant *A. nidulans* strain over-expressing the bZIP transcription factor gene, *rsmA*, which results in a great increase of sterigmatocystin (Yin et al., 2012). RsmA activates the C6 transcription factor AflR, the ST pathway-specific regulatory factor required for transcriptional activation of ST biosynthetic genes. One would expect a benefit to grazers when exposed to *A. nidulans* deficient in the formation of sterigmatocystin. Interestingly, this hypothesis was not supported by Trienens and Rohlfs (2012). Compared with mortality in the presence of sterigmatocystin-producing wild type *A. nidulans*, *Drosophila* larvae did not demonstrate increased survival when confronted with 1-day old *A. nidulans* mutants (Δ*aflR*, Δ*stcJ*, Δ*stcE*, Δ*stcU*) incapable of producing sterigmatocystin. To our surprise, when the insects were exposed to initially 2-days old colonies, larvae suffered even higher mortality on substrate infested with the sterigmatocystin deficient mutant strains (Trienens and Rohlfs, 2012). A similar grazer response was observed when *F. candida* was offered a sterigmatocystin deficient Δ*aflR A. nidulans* mutant: relative to the wild type strains (Rohlfs et al., 2007), the Collembola suffered unusually high mortality and did not reproduce whatsoever (Albert, 2007). Thus, even though an artificial increase in sterigmatocystin biosynthesis enhances the capacity of *A. nidulans* to resist grazing, the loss of this compound does not reduce resistance as one would expect.

In conclusion, sterigmatocystin does not seem to be the major anti-grazer compound in wild type *A. nidulans*. In contrast, sterigmatocystin biosynthesis appears to hamper the ability of *A. nidulans* to develop even better protection against grazers. In search for an explanation of this phenomenon, one could argue that there are only a few key intermediates of the basic metabolic pathways that provide the starting points for the SM pathways. For example, acetyl-CoA is the precursor molecule for compound biosynthesis from the polyketide and the isoprenoid pathway. Possibly, artificial inactivation of the sterigmatocystin pathway leads to higher amounts of acetyl-CoA available for shunting into other pathways which produce more efficient anti-grazer compounds. If this turns out to be an adequate explanation for sterigmatocystin deficient *A. nidulans* being more detrimental to grazers than the wild type, fungi may suffer ecological costs of (high) SM diversity. And perhaps global, e.g., LaeA-dependent, regulatory mechanisms might prove to constrain fungi in activating the optimal combination of pathways for conquering grazers with a less diverse but more effective blend of SMs.

## RESPONSES OF FUNGI TO GRAZERS

It has been well appreciated that fungal chemical phenotypes can vary with abiotic conditions such as light, water, temperature or the availability of nutrients (Schmidt-Heydt et al., 2008; Atoui et al., 2010; Schmidt-Heydt et al., 2010; Nielsen et al., 2011). Also, interactions with bacteria have been established as critical determinants of fungal SM composition (Brakhage and Schroeckh, 2011). Some recent analyses of the chemical responses to animal grazers provide first evidence of an inducible chemical compound-based defense response in fungi, which comprises the biosynthesis of so-called cryptic metabolites.

### EVIDENCE FROM *Aspergillus nidulans*

**FIGURE 1:**
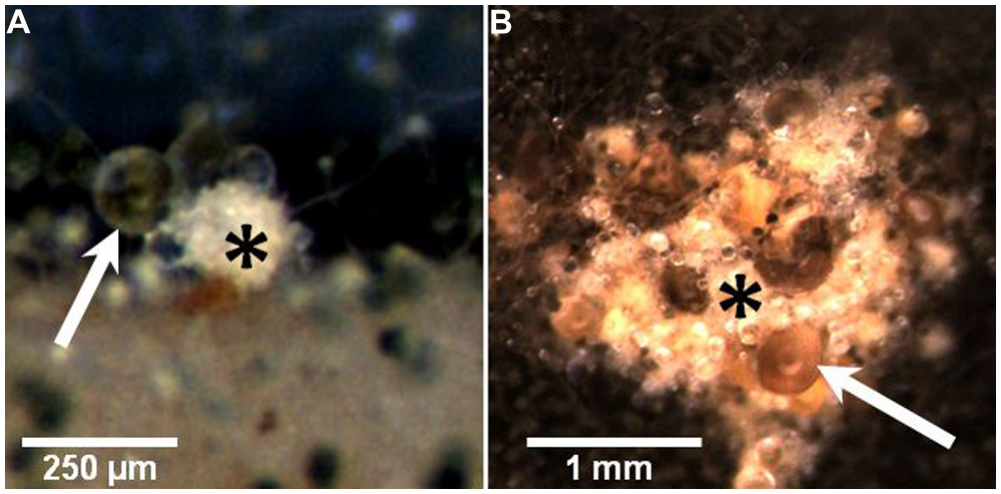
**Images depicting the localized formation of guttation droplets on the sexual fruiting bodies (cleistothecia) of *A. nidulans*, which are possibly involved in mediating protection from grazing by Collembola (see Döll et al., 2013). (A)** To the left of the initial stage (primodium) of a cleistothecium (*asterisk*) a strikingly large droplet (*arrow*) is formed in addition to some smaller ones. The whitish appearance of the primordium is due to layers of so-called Hülle cells which are assumed to nurse and protect the developing ascospores within the cleistothecium. It is not obvious whether the guttation droplets are produced by the Hülle cells or aerial hyphae surrounding the fruiting bodies. **(B)** A cluster of larger cleistothecia (*asterisk*), surrounded by a dark mat of conidia-producing tissue. The cluster is covered by voluminous droplets of light-brown color (*arrow*). Numerous smaller and apparently colorless droplets attached to single aerial hyphae are also visible.

**FIGURE 2:**
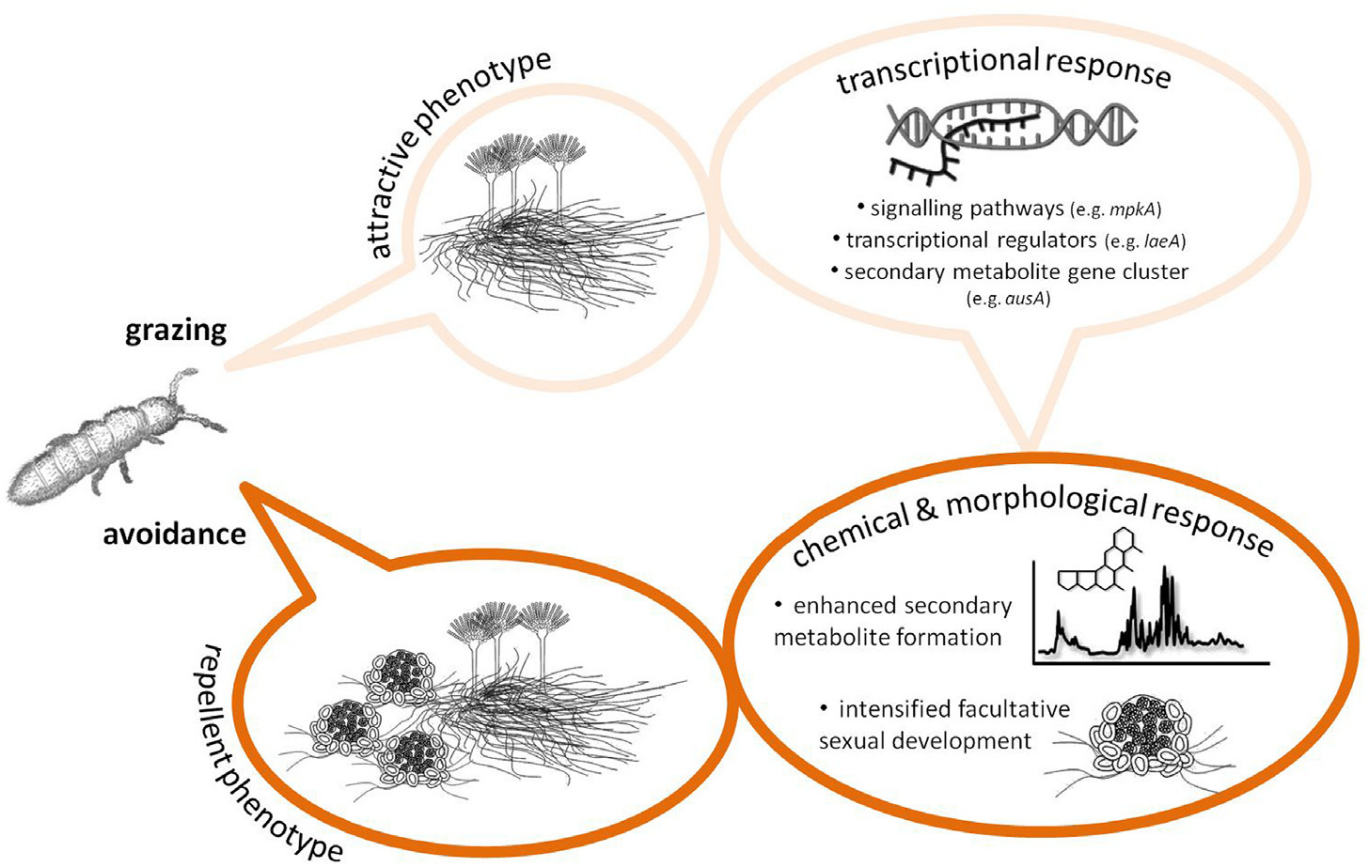
**Scheme summarizing the results of some recent studies demonstrating a grazer-induced defense response in the model fungus *Aspergillus nidulans* [see text and publications by Döll et al. (2013) and Caballero Ortiz et al. (2013) for details].** This scheme may serve as a modifiable blueprint for future studies providing evidence for or against an inducible chemical defense in fungi, add more specific information from other fungal systems, or contribute to general, system-independent properties of fungal chemical responses to grazers, e.g., hormone and pheromone signaling. Although not indicated in detail, it should also be specified how both putative defense compounds affect fungivore behavior and physiology, to be better able to determine the ecological consequences of fungal secondary metabolites (SM) biosynthesis and hence the selective forces that may have, at least in part, shaped fungal SM diversity.

Grazing by Collembola, *F. candida*, was found to induce an increase in the formation of sterigmatocystin, some meroterpenoids (mixed polyketide/terpenoid orign) and emericellamides (mixed polyketide/peptide origin) in *A. nidulans* (Döll et al., 2013). In choice experiments, Collembola preferred un-grazed colonies to grazed, probably due to changes in the volatiles released by damaged colonies (Staaden et al., 2011), and when forced to feed on un-grazed or grazed mold the animals grew slower on previously attacked colonies. Moreover, grazer-challenged *A. nidulans* colonies intensified significantly the formation of sexual fruiting bodies (cleistothecia), which appeared to be the only fungal tissue that was not consumed after prolonged grazing (Döll et al., 2013). Possibly, the positive correlation of the appearance of cleistothecia and the intensive formation of guttation droplets on the sexual fruiting bodies (**Figure 1**) is a means of protecting this valuable tissue from grazers. For example, guttation droplets produced by *Penicillium* and *Stachybotrys* are known to contain high amounts of toxic SMs (Gareis and Gareis, 2007; Gareis and Gottschalk, 2014), yet, no report exists on the SM content in *A. nidulans* guttation droplets exists (and whether it changes under grazer pressure). Nonetheless, at least for *A. nidulans*, combined investment in SM formation and sexual development seems to be a strategy to maintain high fitness in grazer-rich niches (**Figure 2**).

These findings are supported by an experiment that examined the effect of larval *Drosophila* grazing on the expression of *A. nidulans* genes. In the presence of the insect larvae, *A. nidulans* exhibited shifts in the transcriptional activity of many genes, including those involved in signal transduction, hormonal signaling, and SM biosynthesis (Caballero Ortiz et al., 2013); interestingly, *laeA* ranked highest among those genes that were found up-regulated in response to the insects. Comparable with the results of the Collembola-*A. nidulans* experiment described above, grazed wild type colonies were found to kill *Drosophila* fly larvae more rapidly than unchallenged ones. An intriguing finding here was that, in a subsequent experiment, *D. melanogaster* larvae were able to use a Δ*laeA* mutant of otherwise fatal *A. nidulans* as the only available food source that promoted development into adult flies (Caballero Ortiz et al., 2013). That is LaeA-mediated activation of SM formation in response to grazing to a large extent prevents the fungus from being consumed and used as a suitable diet by these facultative fungivores.

Finally, terpenoid compounds that function as hormones in arthropods, the so-called juvenile hormones (JH), have recently been found to be synthesized by *A. nidulans*, in particular under larval fruit fly grazing pressure (Nielsen et al., 2013). And when confronted with a JH over-expression mutant, fly larvae were significantly smaller. Possibly, the anti-grazer defense mechanisms of *A. nidulans* comprise the activity of insect hormone analogs that derange grazer endocrine processes.

Taken together, these findings illustrate the extent to which fungal morphological and chemical properties are affected by dynamic interactions with grazers and thereby reveal a number of new candidate defense pathways that may mediate direct resistance. Considering this chemical diversity of compounds we still lack convincing evidence for the identity of SMs used by fungi to repel and/or harm grazers. Even in this single *A. nidulans* system, it seems possible that more than one master defense compound exists, which are likely to interact with grazers via an array of perhaps equally diverse (patho-) physiological and behavioral mechanisms in the animals.

### EVIDENCE FROM OTHER FUNGI

Using model fungal systems has many obvious advantages, yet there is a strong need to investigate the idea of inducible chemical compound-based resistance in other fungi. Outside the *A. nidulans* system, to the best of my knowledge, there is no good evidence of inducible chemical compound-based resistance in fungi. The study by Bleuler-Martínez et al. (2011) shows the induction of fruiting body lectins (carbohydrate-binding proteins) by nematode worms piercing hyphae and sucking in the cytoplasmatic content of *Coprinopsis cinerea*. In spite of this clear demonstration of an induced fungal response, it remains to be tested whether lectins provide an effective protection against the nematode or other grazers, since lectin toxicity has only been tested against non-fungivores (Bleuler-Martínez et al., 2011; Schubert et al., 2012; Žurga et al., 2014). Relevant to this discussion is the finding by Balogh et al. (2003), who did not find a positive influence of a lectin-deficient *Arthrobotrys oligospora* mutant on grazing by *F. candida*.

These very first studies illustrate the significance of grazing in determining the chemical profile of fungi but also the complexities and ambiguities involved in establishing a conceptually sound and direct connection between SM formation and fungal fitness. Therefore, separating functionally relevant from irrelevant chemical responses of fungi to grazing will be of utmost importance for the correct annotation of putative fungal defense traits that mediate protection from grazers. For example, genome-wide gene expression analyses should follow to reveal fungal responses to grazing the abovementioned studies may have overlooked.

## CONCLUSION

We are only beginning to appreciate fungal chemical dynamics under varying ecological conditions, and we should investigate deeper the full repertoire, kinetics, and sites of secondary metabolite biosynthesis in response to grazers to be able to update and rearrange the lists of putative defense compounds (in *A. nidulans* and other fungi) on the basis of inducible reactions. Carefully conducted experiments with model grazers (e.g., Collembola, *Drosophila* larvae, nematodes, etc.) exposed to purified compounds will shed light on the mode of action in fungivores, e.g., whether fungal chemicals decrease or suppress the feeding response, act as acute toxins that interfere with intermediary metabolism or cellular functions, or cause chronic tissue and organ malfunctions which ultimately lead to impaired development and reduced fecundity. Finally, a large fraction of fungal SMs likely have evolved to allow effective control of and/or communication with other microbes in their environment, and we should thus think about the possibility that it is not the grazer itself that is directly affected by specific metabolites, but the endogenous microorganisms fungivores require for the control of their immune system, food breakdown, and detoxification. Thus, in order to fully appreciate the complete anti-grazer potential of SM biosynthesis in fungi, we need to combine their inducible dynamics with the influence of these compounds on grazer behavior, physiology, and multi-species interactions in their environment.

## Conflict of Interest Statement

The author declares that the research was conducted in the absence of any commercial or financial relationships that could be construed as a potential conflict of interest.
